# Serial changes in left ventricular myocardial deformation in sepsis or septic shock using three-dimensional and two-dimensional speckle tracking echocardiography

**DOI:** 10.3389/fcvm.2022.925367

**Published:** 2022-08-04

**Authors:** Xiaojun Yan, Yuman Li, Juanjuan Liu, Ting Zhou, Yi Zhou, Wei Sun, Chenchen Sun, Jing Ma, Li Zhang, You Shang, Mingxing Xie

**Affiliations:** ^1^Department of Ultrasound Medicine, Union Hospital, Tongji Medical College, Huazhong University of Science and Technology, Wuhan, China; ^2^Clinical Research Center for Medical Imaging in Hubei Province, Wuhan, China; ^3^Hubei Province Key Laboratory of Molecular Imaging, Wuhan, China; ^4^Department of Critical Care Medicine, Union Hospital, Tongji Medical College, Huazhong University of Science and Technology, Wuhan, China; ^5^Tongji Medical College and Wuhan National Laboratory for Optoelectronics, Huazhong University of Science and Technology, Wuhan, China

**Keywords:** sepsis, septic shock, myocardial deformation, three-dimensional speckle tracking echocardiography, two-dimensional speckle tracking echocardiography

## Abstract

**Background:**

This study aimed to investigate the serial changes in left ventricular (LV) myocardial deformation in patients with sepsis using three-dimensional (3D) and two-dimensional (2D) speckle tracking echocardiography (STE).

**Methods:**

In this single-center, prospective, and observational study, we included 59 patients diagnosed with sepsis or septic shock in the intensive care unit and 40 healthy controls. Left ventricular ejection fraction (LVEF), left ventricular global longitudinal strain (GLS), and global circumferential strain (GCS) assessed by 3D STE and 2D STE were obtained on the first, third, fifth, seventh to the tenth day after sepsis or septic shock.

**Results:**

In patients with sepsis or septic shock, 3D and 2D LVEF were not different at each time point. GLS and GCS obtained by 3D STE and 2D STE decreased on the first day compared with the healthy group (all *P* < 0.01). Compared with the values on the first day, GLS and GCS further decreased on the third day, while 3D and 2D LVEF did not differ. 3D and 2D STE strains were lowest on the third day and gradually improved on the seventh to the tenth day compared with values on the third day. When compared with values on the first day, 3D and 2D GLS gradually improved on the seventh to the tenth day, whereas 3D and 2D GCS on the seventh to the tenth day was not different. Although 3D and 2D STE strains were significantly increased on the seventh to the tenth day, they were not fully recovered to normality.

**Conclusion:**

Although patients with sepsis or septic shock demonstrated gradual improvements in 3D and 2D STE parameters during the ten-day period, LV myocardial strain was not fully recovered to normality by the seventh to the tenth days. 3D and 2D strain imaging, used as a helpful tool for monitoring the evolution of myocardial deformation, can provide clinicians with a useful additional imaging parameter.

## Introduction

Sepsis is associated with high morbidity and mortality in contemporary intensive care units worldwide (1, 2). A growing body of literature has recognized the adverse impact of left ventricular (LV) dysfunction associated with sepsis and septic shock on mortality (3–6). Therefore, early identification of LV dysfunction plays a pivotal role in treating circulatory impairment and stratifying risk in patients with sepsis (7). LV function develops dynamically when the circulatory system is disturbed for 10 days. Therefore, accurate monitoring of serial changes in LV function provides clinics with vital information to guide the optimal treatment of patients. Despite increasing awareness of the importance of monitoring for LV dysfunction in patients with sepsis or septic shock, the serial evolution of LV function has not been well characterized.

Left ventricular ejection fraction (LVEF) is a common echocardiographic measurement for assessing the global systolic function of the left ventricle (8, 9). However, in the early period of sepsis-induced cardiac dysfunction, LVEF is often not susceptible to the slight injury of myocardial movement and is likely to depend on the patients' heart rate and various rehydration treatment loads (10). Thus, it is not an ideal index for detecting early, subtle changes in myocardial function. In recent years, two-dimensional speckle tracking echocardiography (2D STE) has become a powerful tool for accurately quantifying cardiac mechanics owing to its less load- and angle-dependency and has been demonstrated to be more sensitive in detecting subclinical cardiac dysfunction than LVEF (11). Currently, limited data exist regarding the changes in LV strain assessed by 2D STE during the first 3 days in patients with sepsis or septic shock (12, 13). However, 2D STE is hindered by the 2D plane and out-of-plane movement of the speckles, ignoring the characteristics of three-dimensional (3D) cardiac wall motion. More recently, 3D STE is rapidly becoming an essential technique in accurately and comprehensively evaluating myocardial function owing to overcoming the limitations of 2D STE by analyzing the regional wall motion of the entire left ventricle (14). Although 3D STE is theoretically superior to 2D STE for quantifying LV deformation, it needs clinical validation. Until now, the application value of 3D STE in patients with sepsis or septic shock has not been established.

Therefore, this study aimed to investigate the serial changes in the 3D and 2D strains of the left ventricle in patients with sepsis or septic shock during a ten-day period.

## Materials and Methods

### Study design and participants

This prospective observational study was performed at Union Hospital, Tongji Medical College, Huazhong University of Science and Technology, Wuhan, China, between December 2020 and December 2021. Patients (≥18 years old) who met the sepsis and septic shock criteria defined by the international guidelines for the management of sepsis and septic shock in 2016 were included in the study (8). Patients with known myocardial dysfunction, severe valvular heart disease, arrhythmia, myocardial infarction, or lacking good 2D/3D echo images were excluded. Known myocardial dysfunction was defined as LVEF <50%. Severe valvular heart disease was defined as severe valvular stenosis and/or severe regurgitation, and patients who had previously undergone valvular intervention. A total of 113 patients with sepsis or septic shock and 40 healthy subjects in our hospital were scheduled for 3D and 2D echocardiograms. The study protocols were approved by the institutional Ethical Committee of the Union hospital, Tongji Medical College, Huazhong University of Science and Technology, Wuhan, China (2021-S047). The patients' family members signed the informed consent.

### Clinical data

Demographic data of participants included age, sex, body mass index (BMI), and body surface area (BSA). Past medical history included hypertension, diabetes mellitus, coronary artery disease, malignancy, chronic liver disease, and chronic kidney disease. The sources of infection included the hepato-biliary-pancreas, the lungs, and the gastrointestinal tract. Laboratory findings were collected. The severity of illness was assessed according to the Acute Physiology and Chronic Health Evaluation (APACHE-II) and sequential organ failure assessment (SOFA) scores. Norepinephrine is the first-choice vasopressor therapy (used to target a mean arterial pressure of 65 mmHg or more). All patients were treated according to international guidelines for the treatment of septic shock at the discretion of the treating clinicians after initial resuscitation.

### Conventional echocardiography

All echocardiographic examinations were performed using Philips echocardiographic systems (EPIQ 7C; S5-1, X5-1 transducer; Philips Healthcare, Andover, MA, USA). We separated echocardiographic data based on time intervals, namely, first, third, fifth, and seventh to the tenth day after being diagnosed with sepsis or septic shock after ICU admission. In addition, electrocardiographic gating was performed during the echocardiographic image acquisition. LA volume was assessed with the biplane method of disks from the apical four- and two-chamber views (15). Doppler mitral valve peak early (E) and late (A) diastolic velocities and E/A velocity ratio were measured from the apical four-chamber view. The mean value of early diastolic mitral annular tissue velocity and left ventricular lateral wall tissue velocity (e') were measured by tissue Doppler imaging. Two-dimensional LVEF was obtained using the two-biplane Simpson method based on end-systolic/diastolic LV volumes. All 2D and 3D echocardiographic images were acquired according to the published guidelines of the American Society of Echocardiography by two experienced operators who were blinded to the clinical characteristics of the participants (11).

### Two-dimensional speckle-tracking analysis

2D STE analysis was performed using vendor-independent 2D speckle-tracking software (2D Cardiac Performance Analysis Ver 1.3, TomTec, Germany) based on the previously described method (16). 2D images with ten consecutive cardiac cycles were stored for 2D STE analysis. The clearest cardiac cycle was selected for 2D STE analysis. LV longitudinal strain was measured in the apical four-chamber, two-chamber, and long-axis views. LV circumferential strain was measured by endocardial tracing in the basal, middle, and apical levels of LV short-axis views. After the software automatically tracked the speckles in the myocardium frame-by-frame basis during the entire cardiac cycle, the software provided regional strain curves. The peak regional strain value was obtained. 2D global longitudinal strain (GLS) and 2D global circumferential strain (GCS) were calculated as the peak strain values from the averaged strain curves generated from 16 segmental strain curves. Adequacy of tracking was verified visually, and if the tracking seemed incorrect, manual adjustment of the endocardial border was performed.

### Three-dimensional speckle-tracking analysis

3D full-volume data were acquired with the use of a 3D matrix-array transducer from the LV-focused apical 4-chamber view; the fan angle and depth were adjusted to cover the whole region of interest. 3D images with ten consecutive cardiac cycles were stored for 3D STE analysis. 3D full-volume datasets were analyzed by a vendor-independent 3D speckle-tracking software (3D Cardiac Performance Analysis Ver 1.3, TomTec, Germany). The investigator selected the clearest cardiac cycle for full volume tracking. In the apical four and two-chamber views of the end-diastolic frame, the apical point of the LV and the center of the mitral annular line were selected to set the largest LV apical long-axis dimensions, and the corresponding landmarks of the aortic annulus were identified in the apical three-chamber view. The workstation tracked the LV endocardium automatically, and manual adjustment was performed in case of unsatisfactory tracking. Ultimately, the 3D LV end-diastolic volume (EDV), end-systolic volume (ESV), LVEF, and the myocardial strain generated automatically. LVGLS and LVGCS were calculated as the average peak systolic longitudinal and circumferential strain of all 16 LV segments.

### Reproducibility

To evaluate the reproducibility of the 3D STE and 2D STE measurements, 30 subjects were randomly selected, and the measurements were repeated. For intraobserver variability, analysis of the first 3D STE and 2D STE data set was repeated 2–4 weeks later by the same primary investigator. For interobserver variability, the data set was analyzed by two blinded investigators.

### Statistical analysis

Continuous variables were expressed as mean ± SD for normally distributed data or median [interquartile range (IQR)] for abnormally distributed variables. Categorical variables were expressed as numbers (percentages). For comparison between groups, the Kruskal Wallis H and repeated-measures analysis of variance (ANOVA) were used for continuous variables. Correlations between continuous variables were evaluated with Pearson's correlation coefficients. Bias and limits of agreement (LOA) between two different measurements were evaluated using the Bland–Altman analysis. To compare with the healthy group, an independent sample *t*-test was used. The interobserver and intraobserver variability of the 3D and 2D STE parameters were assessed by the intraclass correlation coefficients (ICCs) and the Bland–Altman analyses. The data were analyzed with IBM SPSS Statistics for Windows (Version 26.0, IBM Corp Armonk, NY, USA). A two-sided *p*-value < 0.05 was considered significant.

## Results

### Clinical characteristics

**Figure 1:**
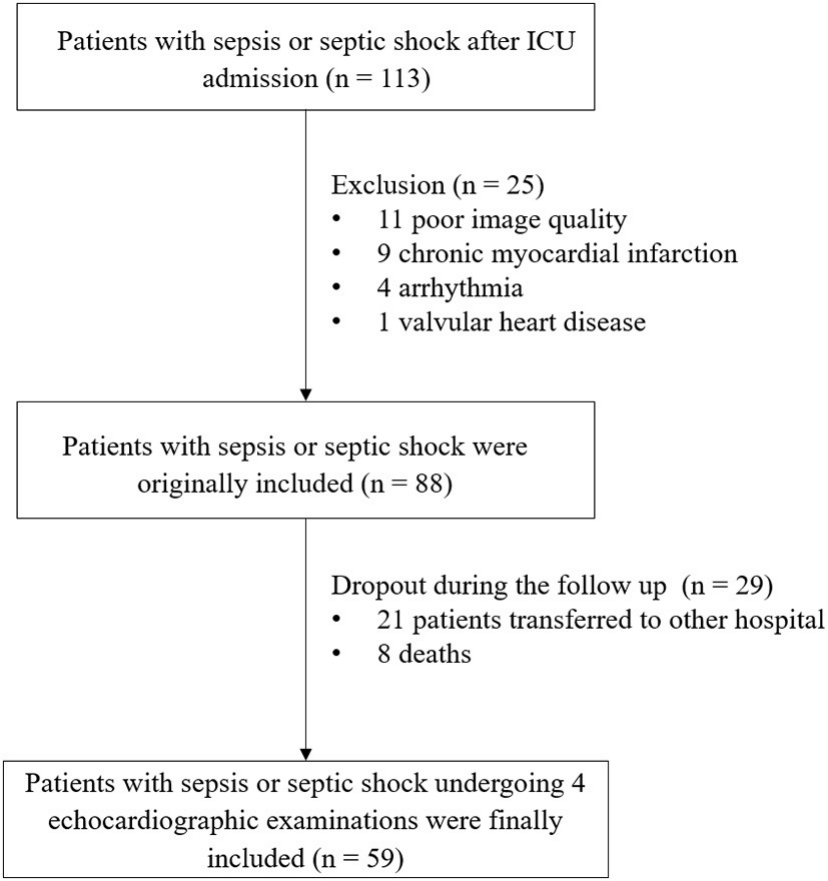
Study flowchart.

Tables 1, 2 summarize the baseline clinical characteristics of the patients. Among the 113 patients, after the exclusion of 11 participants with inadequate echocardiography images, nine with myocardial infarction, four with arrhythmia, one with severe valvular heart disease, and an additional 29 patients who were lost to follow-up during the study period, 59 individuals were included (Figure 1). The median age of patients with sepsis or septic shock was 56 (48 to 69) years, and 35 (59.3%) patients were men. During the period of medical treatment, 34 (57.6%) patients were given norepinephrine, six (10.2%) patients dobutamine, seven (11.9%) patients levosimendan, and four (6.7%) patients amiodarone. Most patients had organ function damage, with laboratory data showing the abnormalities within 10 days. The APACHE II score, heart rate, oxygenation index, uric acid, D-dimer, and CK-MB decreased gradually, (all, *P* < 0.05).

**Table 1:** Demographics and baseline characteristics of patients.

**Characteristics**	**Total (*n* = 59)**
**Demographics**	
Age, years, median (IQR)	56 (48 to 69)
Men, *n* (%)	35 (59.3)
BMI, kg/m^2^, median (IQR)	22.4 (19.5 to 25.3)
BSA, (m^2^), median (IQR)	1.7 (1.6 to 1.9)
**Past medical history**	
Hypertension, *n* (%)	8 (13.6)
Diabetes mellitus, *n* (%)	7 (11.9)
Coronary Heart Disease, *n* (%)	3 (5.1)
Malignancy, *n* (%)	3 (5.1)
Chronic pulmonary disease, *n* (%)	21 (35.6)
Chronic kidney disease, *n* (%)	20 (33.9)
**Infection site**	
Gastrointestinal, *n* (%)	3 (5.1)
Hepato-biliary-pancreas, *n* (%)	24 (40.7)
Pulmonary, *n* (%)	16 (27.1)
Unknow, *n* (%)	7 (11.9)
**Medicine**	
Norepinephrine treatment, *n* (%)	34 (57.6)
Dobutamine treatment, *n* (%)	6 (10.2)
Levosimendan treatment, *n* (%)	7 (11.9)
Amiodarone treatment, *n* (%)	4 (6.7)

*Data are presented as medians (lower quartile to upper quartile) and number (n) of patients (%)*.

**Table 2:** Intensive care measurements and vital signs over time in patients.

**Characteristics**	**1^st^ Day (*n =* 59)**	**3^rd^ Day (*n =* 59)**	**5^th^ Day (*n =* 59)**	**7^th^-10^th^ Day (*n =* 59)**	***P***
APACHE II score	29 (25 to 32)	19 (6 to 27)^⋆^	18 (7 to 27)^⋆^	21 (8 to 27)^⋆^	<0.001
SOFA score	11 (9 to 14)	13 (11 to 15)	13 (10 to 15)	12 (11 to 14)	0.418
Systolic pressure (mmHg)	121 (110 to 131)	120 (102 to 129)	126 (111 to 133)	125 (101 to 137)	0.444
Diastolic pressure (mmHg)	62 (56 to 67)	60 (55 to 65)	62 (53 to 68)	64 (54 to 68)	0.470
Heart rate, beats per min	99 (81 to 117)^⋆^	89 (81 to 104)^⋆^	95 (83 to 106)	94 (82 to 102)	0.026
Respiratory rate, beats per min	20 (16 to 23)	18 (16 to 20)	18 (15 to 22)	20 (17 to 22)	0.523
Lactate concentration (mmol/L)	2.0 (1.4 to 3.1)	1.5 (1.1 to 2.0)	1.5 (1.0 to 2.3)	1.4 (0.8 to 2.1)	0.307
Oxygen saturation (SPO2)	98.0 (96.0 to 99.5)	99.0 (96.0 to 100.0)	98.0 (96.0 to 99.0)	98.0 (95.8 to 99.0)	0.381
Oxygenation index	281.0 (212.0 to 328.0)	261.0 (183.0 to 325.0)	308.5 (225.5 to 376.5)	246.0 (194.8 to 286.9)^‡^	0.020
Total volume of fluids administered (ml/kg)	53.2 (41.2 to 83.0)	57.3 (50.2 to 92.2)	63.4 (44.9 to 99.1)	62.0 (39.3 to 120.0)	0.343
Calcium (mmol/L)	2.1 (1.8 to 2.2)	2.0 (1.9 to 2.2)	2.0 (1.9 to 2.2)	2.1 (1.9 to 2.2)	0.612
Phosphorus (mmol/L)	1.0 (0.7 to 1.2)	0.9 (0.7 to 1.1)	1.1 (0.7 to 1.5)	1.0 (0.8 to 1.2)	0.192
Magnesium (mmol/L)	0.8 (0.7 to 0.9)	0.8 (0.7 to 0.9)	0.8 (0.7 to 0.9)	0.9 (0.8 to 0.9)	0.353
Urea Nitrogen	13.6 (8.0 to 21.1)	12.8 (7.3 to 19.5)	15.6 (11.1 to 28.4)^†^	12.5 (8.1 to 20.4)^⋆‡^	0.064
Creatinine (umol/L)	136.5 (60.8 to 230.3)	77.8 (49.2 to 167.1)	121.0 (62.3 to 177.1)	120.7 (49.7 to 266.9)	0.101
Uric acid (ummol/L)	257.4 (154.5 to 417.4)	153.1 (103.2 to 273.7)^⋆^	233.3 (128.8 to 373.2)	203.0 (131.3 to 304.6)	0.001
Glomerular filtration rate	36.8 (18.6 to 92.5)	71.0 (30.4 to 105.5)	51.8 (31.6 to 100.6)	56.0 (22.8 to 104.0)	0.119
D-dimer (ug/ml)	8.0 (4.7 to 16.9)	5.8 (3.0 to 8.8)^⋆^	5.1 (2.8 to 9.4)^⋆^	3.7 (2.3 to 8.1)^⋆^	0.004
PCT (ng/ml)	5.6 (2.2 to 47.2)	6.7 (0.9 to 37.4)	4.8 (0.9 to 29.2)	3.9 (1.5 to 16.6)	0.221
C-reactive protein (mg/L)	123.0 (67.9 to 184.5)	95.8 (42.4 to 156.5)	105.0 (58.4 to 166.5)	100.5 (54.5 to 168.0)	0.211
Aspartate aminotransferase (U/L)	42.0 (23.0 to 96.3)	41.5 (24.8 to 87.8)	39.0 (27.5 to 79.0)	39.0 (26.0 to 69.0)	0.229
Creatine Kinase (U/L)	134.0 (56.0 to 341.0)	121.0 (37.0 to 364.0)	78.0 (33.0 to 369.0)	54.0 (22.0 to 191.0)	0.535
Lactate dehydrogenase (U/L)	347.0 (204.0 to 785.0)	312.0 (237.0 to 711.0)	381.0 (257.5 to 561.5)	348.0 (258.3 to 460.5)	0.194
CK-MB (U/L)	2.3 (1.0 to 7.7)	1.5 (0.5 to 4.6)	1.0 (0.5 to 5.0)	1.1 (0.6 to 6.6)	0.754
High sensitivity troponin T (ug/L)	53.0 (12.8 to 540.8)	77.7 (19.5 to 525.1)	52.9 (13.4 to 214.0)	57.4 (13.9 to 181.9)	0.741
NT-proBNP (ng/L)	240.3 (107.5 to 733.0)	502.6 (168.7 to 1,563.0)	341.3 (160.9 to 1,308.3)	266.2 (91.9 to 683.7)	0.414
Total norepinephrine treatment (mg)	8.0 (4.0 to 9.0)	6.0 (4.0 to 9.5)	8.0 (4.0 to 16.0)	6.0 (4.0 to 16.0)	0.706

*Data are presented as medians (lower quartile to upper quartile) and change over time. ^⋆^Comparison to 1^st^ day. ^†^Comparison to 3^rd^ day. ^‡^Comparison to 5^th^ day. a two-sided P-value < 0.05 was considered to indicate statistical significance. APACHE II, acute physiology and chronic health evaluation; SOFA, sequential organ failure assessment; PCT, procalcitonin; CK-MB, creatine kinase isoenzymes; NT-proBNP, amino-terminal pro-brain natriuretic peptide*.

### Serial changes in LV function

Echocardiographic characteristics over time in patients are displayed in Table 3. 2D LVEF did not differ among each time point assessed (*P* = 0.100). Likely, 3D LV volumes and 3D LVEF were also not different at each time point accessed during the ten-day period (*P* = 0.146).

**Table 3:** Echocardiographic Parameters by 2D and 3D Measurements over time in patients.

	**1^st^ Day (*n* = 59)**	**3^rd^ Day** **(*n* = 59)**	**5^th^ Day (*n* = 59)**	**7^th^-10^th^ Day (*n* = 59)**	***P***
**Conventional echo**					
LA volume (ml)	35.1 ± 4.7	34.4 ± 4.1	34.4 ± 4.5	33.9 ± 4.3	0.632
LV diameter (mm)	47.5 ± 6.8	47.2 ± 5.5	45.9 ± 5.1	46.9 ± 4.6	0.481
LVEDVI (mL/m^2^)	63.5 ± 17.9	53.5 ± 19.4	60.7 ± 22.7	67.9 ± 21.8^†^	0.029
LVESVI (mL/m^2^)	27.0 ± 9.0	24.4 ± 10.4	26.3 ± 13.2	27.3 ± 11.1	0.595
SVI (mL/m^2^)	36.5 ± 10.2	29.1 ± 11.9^⋆^	34.4 ± 13.9	40.7 ± 15.9^⋆†^	0.001
LVEF (%)	57.6 ± 5.4	55.2 ± 6.4	57.3 ± 7.8	59.7 ± 7.5	0.100
E/A ratio	1.0 ± 0.4	1.1 ± 0.5	1.0 ± 0.3^†^	1.1 ± 0.3	0.364
E/e' ratio	10.9 ± 5.6	10.5 ± 5.6	10.5 ± 5.3	9.9 ± 3.9	0.817
**2D-STE**					
Frame rate (frame /sec)	52.0 ± 5.0	51.0 ± 5.0	51.0 ± 4.0	50.0 ± 5.0	0.973
2D-LVGLS (%)	–16.0 ± 2.4	–14.1 ± 4.6^⋆^	–16.0 ± 4.1^†^	–17.9 ± 3.3^⋆†‡^	<0.001
2D-LVGCS (%)	–18.2 ± 4.1	–16.1 ± 5.4^⋆^	–18.2 ± 4.5^†^	–19.1 ± 4.7^†^	0.002
**3D-STE**					
Frame rate (volumes /sec)	24.0 ± 3.0	25.0 ± 3.0	24.0 ± 3.0	24.0 ± 4.0	0.935
3D-LVGLS (%)	–15.3 ± 2.7	–13.8 ± 3.6^⋆^	–16.1 ± 4.0^†^	–18.0 ± 2.8^⋆†‡^	<0.001
3D-LVGCS (%)	–19.6 ± 4.7	–16.9 ± 4.6^⋆^	–19.7 ± 5.8^†^	–20.8 ± 4.4^†^	0.001
3D-LVEDVI (mL/m^2^)	54.4 ± 18.9	52.5 ± 13.5	56.9 ± 17.6	61.3 ± 22.7	0.149
3D-LVESVI (mL/m^2^)	25.9 ± 10.4	23.4 ± 7.3	24.9 ± 13.9	26.2 ± 11.0	0.581
3D-SVI (mL/m^2^)	28.5 ± 11.8	29.1 ± 7.9	31.9 ± 11.8	35.1 ± 16.7	0.055
3D-LVEF (%)	55.6 ± 4.7	52.4 ± 9.3	56.5 ± 7.2	57.1 ± 6.3	0.146

*Data are presented as the mean ± SD for continuous variables. ^⋆^Comparison to 1^st^ day. ^†^Comparison to 3^rd^ day. ^‡^Comparison to 5^th^ day. A two-sided P-value < 0.05 was considered to indicate statistical significance. LA, left atrium; LV, left ventricular; LVEF, left ventricle ejection fraction; LVEDVI, left ventricular end-diastolic volume index; LVESVI, left ventricular end-systolic volume index; SVI, stroke volume index; E, early diastolic inflow velocity; A, late diastolic inflow velocity; E/e', early mitral inflow to mitral annular motion velocity ratio; GLS, global longitudinal strain; GCS, global circumferential strain*.

**Figure 2:**
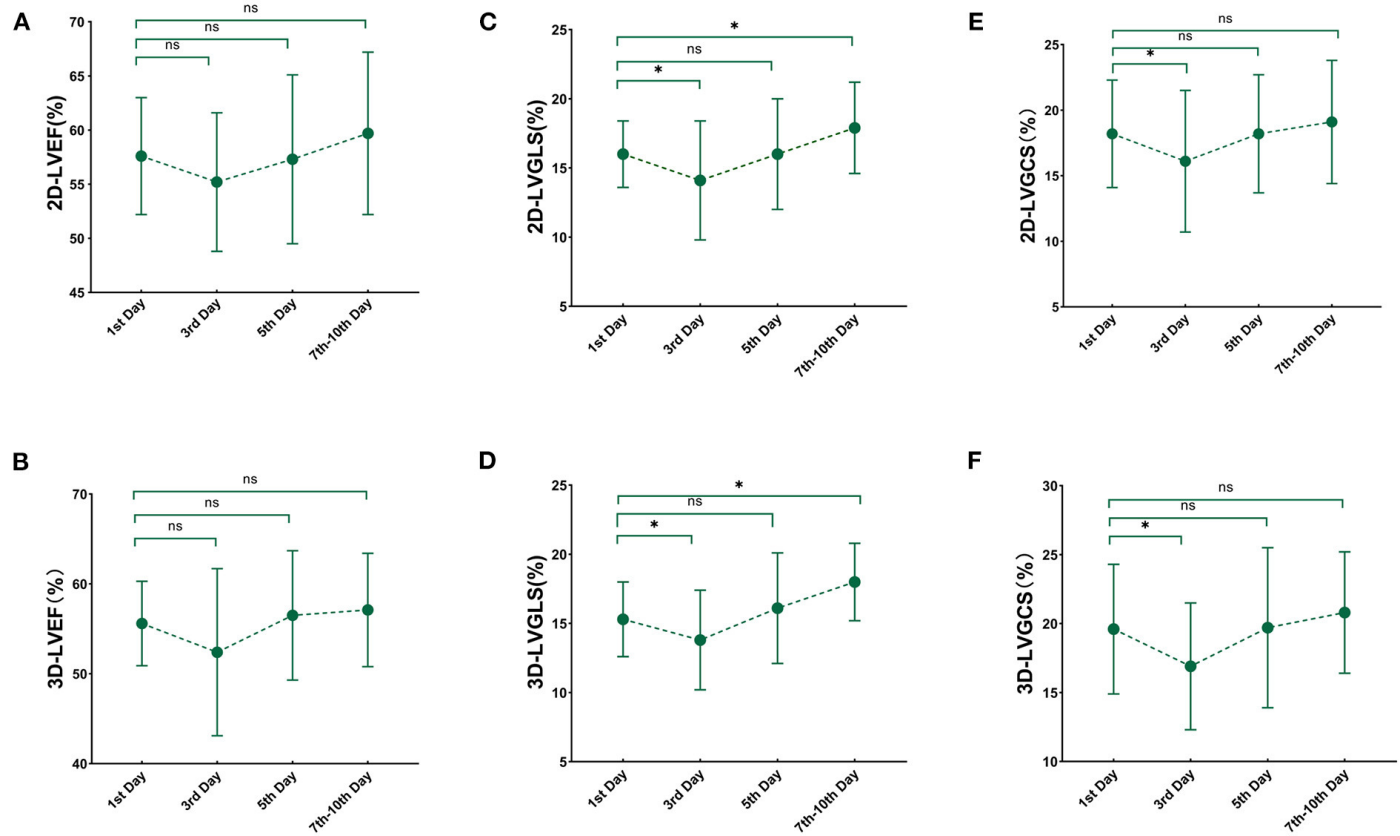
Serial changes in 3D and 2D echocardiography paremeters over time. Line chart for 3D and 2D echocardiography at each time point after sepsis and septic shock. **(A)** 2D-LVEF; **(B)** 3D- LVEF; **(C)** 2D-LVGLS; **(D)** 3D-LVGLS; **(E)** 2D-LVGCS; **(F)** 3D-LVGCS. LVEF, left ventricular ejection fraction; GLS, global longitudinal strain; GCS, global circumferential strain; 2D, two-dimensional; 3D, three-dimensional. *'< 0.05 comparison to 1st day.

3D and 2D LV myocardial deformation showed a significant improvement within 10 days (3D LVGLS, *P* < 0.001; 3D LVGCS, *P* = 0.001; 2D LVGLS, *P* < 0.001; 2D LVGCS, *P* = 0.002). Compared with the values on the first day, GLS and GCS assessed by 3D and 2D STE further decreased on the third day, while 3D and 2D LVEF did not differ (Figure 2). 3D and 2D STE strains were lowest on the third day, which then gradually improved on the seventh to the tenth day (comparison of values between the third and the seventh to the tenth day: 3D LVGLS, −13.8 ± 3.6% vs. −18.0 ± 2.8%, *P* < 0.001; 3D LVGCS, −16.9 ± 4.6% vs. −20.8 ± 4.4%, *P* = 0.001; 2D LVGLS, −14.1 ± 4.6% vs. −17.9 ± 3.3%, *P* < 0.001; 2D LVGCS, −16.1 ± 5.4% vs. −19.1 ± 4.7%, *P* = 0.002). Compared with the values on the first day, 3D and 2D GLS on the seventh to the tenth day increased significantly, whereas 3D and 2D GCS on the seventh to the tenth day did not attain a significant difference.

LV strain values obtained by 3D and 2D STE on the first and the seventh to the tenth day compared with healthy controls are presented in Table 4. Compared with the healthy strain values, 3D and 2D STE strains were decreased on the first day (3D LVGLS, −15.3 ± 2.7% vs. −22.2 ± 2.0, *P* < 0.01; 3D LVGCS, −19.6 ± 4.7% vs. −31.0 ± 3.7, *P* < 0.01; 2D LVGLS, −16.0 ± 2.4% vs. −23.5 ± 4.2, *P* < 0.01; 2D LVGCS, −18.2 ± 4.1% vs. −32.0 ± 5.4, *P* < 0.01) and not fully recovered to normality on the seventh to the tenth day (3D LVGLS, −18.0 ± 2.8 vs. −22.2 ± 2.0, *P* < 0.01; 3D LVGCS, −20.8 ± 4.4 vs. – 31.0 ± 3.7, *P* < 0.01; 2D LVGLS, −17.9 ± 3.3 vs. −23.5 ± 4.2, *P* < 0.01; 2D LVGCS, −19.1 ± 4.7 vs. −32.0 ± 5.4, *P* < 0.01).

**Table 4:** 2D- and 3D-STE strain in patients compared with healthy controls.

**Ventricular function parameters**	**Healthy group (*n* = 40)**	**1^st^ Day** **(*n* = 59)**	***P***	**7^th^-10^th^ Day** **(*n* = 59)**	***P***
2D-LVGLS (%)	–23.5 ± 4.2	–16.0 ± 2.4	<0.01	–17.9 ± 3.3	<0.01
2D-LVGCS (%)	–32.0 ± 5.4	–18.2 ± 4.1	<0.01	–19.1 ± 4.7	<0.01
3D-LVGLS (%)	–22.2 ± 2.0	–15.3 ± 2.7	<0.01	–18.0 ± 2.8	<0.01
3D-LVGCS (%)	–31.0 ± 3.7	–19.6 ± 4.7	<0.01	–20.8 ± 4.4	<0.01

*LVEF, LV ejection fraction; GLS, global longitudinal strain; GCS, global circumferential strain. Data are expressed as mean ± SD*.

### The correlation and agreement between 3D STE and 2D STE parameters

**Figure 3:**
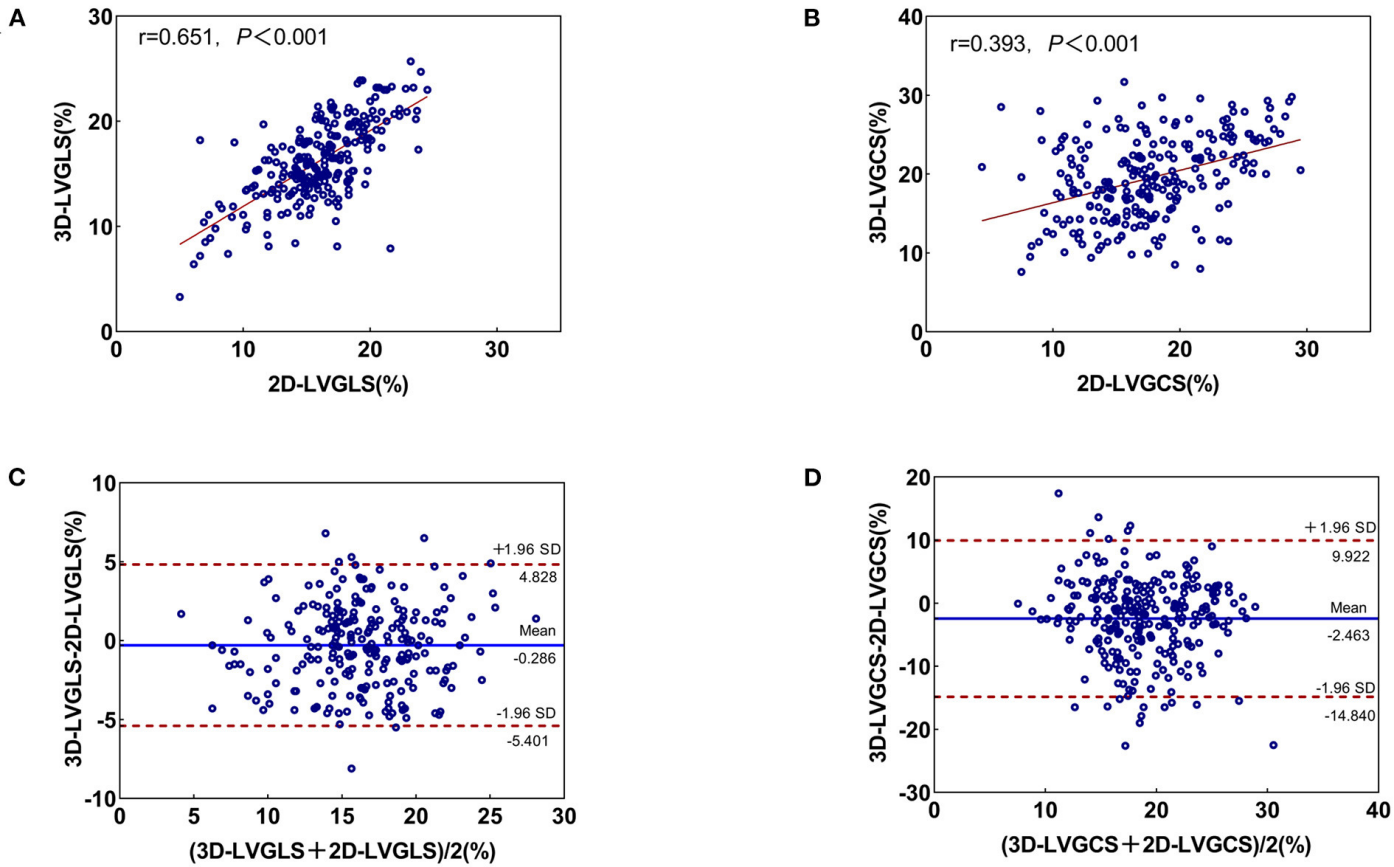
Correlation and Bland-Altman analysis for 3D- and 2D-STE indices. **(A)** The correlation between 2D-LVGLS and 3D-LVGLS; **(B)** The consistency between 2D-LVGLS and 3D-LVGLS; **(C)** The correlation between 2D-LVGCS and 3D-LVGCS; **(D)** The consistency between 2D-LVGCS and 3D-LVGCS. GLS, global longitudinal strain; GCS, global circumferential strain; 2D, two-dimensional; 3D, three-dimensional.

The correlation and agreement between 3D STE and 2D STE parameters are shown in Figure 3. 3D LVGLS had a stronger correlation with 2D LVGLS than that of 3D LVGCS with 2D LVGCS (0.651 vs. 0.393, *P* < 0.001). Furthermore, the bias values comparing 3D LVGLS with 2D LVGLS were lower than those of 3D LVGCS with 2D LVGCS (bias: −0.286 and −2.463).

### Reproducibility

Interobserver and intraobserver variability of the 3D and 2D STE parameters are presented in Table 5. LV strain obtained by 3D and 2D STE showed excellent reproducibility, as evidenced by the high ICC, small bias, and narrow LOA. Intraobserver and interobserver variability of LV strain measured using the 3D and 2D methods were low (all CVs <10%).

**Table 5:** Intraobserver and interobserver reproducibility for 2D- and 3D-STE parameters.

	**CV, %**	**ICC (95% CI)**	**Bias**	**Limits of agreement**
**Intraobserver (*****n*** **=** **30)**				
2D-LVGLS (%)	3.2	0.97 (0.89–0.99)	–0.6	–2.5 to 1.2
2D-LVGCS (%)	5.2	0.97 (0.77–0.99)	–0.9	–2.9 to 1.0
3D-LVGLS (%)	8.2	0.79 (0.61–0.90)	–0.7	–6.4 to 4.9
3D-LVGCS (%)	9.9	0.97 (0.77–0.99)	–1.8	–4.1 to 0.4
**Interobserver (*****n*** **=** **30)**				
2D-LVGLS (%)	7.5	0.91 (0.73–0.97)	–1.1	–4.2 to 2.0
2D-LVGCS (%)	5.7	0.96 (0.84–0.99)	–1.0	–3.5 to 1.6
3D-LVGLS (%)	4.8	0.93 (0.86–0.97)	–0.1	–3.5 to 3.3
3D-LVGCS (%)	8.7	0.91 (0.34–0.97)	–1.7	–4.6 to 1.1

## Discussion

To the best of our knowledge, this is the first comprehensive observation of serial changes in left ventricular 3D and 2D strain in patients with sepsis or septic shock. The main findings of this study are as follows: (1) Compared with the values on the first day, GLS and GCS assessed by 3D and 2D STE further decreased on the third day, while 3D and 2D LVEF did not differ. (2) 3D and 2D STE strains were lowest on the third day and gradually improved by the seventh to the tenth day in the whole study population but did not fully recover to normality. (3) Compared with the values on the first day, 3D and 2D GLS were significantly increased on the seventh to the tenth day, whereas there was no significant difference in 3D and 2D GCS.

### 2D-STE in patients with sepsis or septic shock

Cardiac dysfunction caused by sepsis, usually defined as sepsis-induced cardiomyopathy, occurs in between 40 and 60% of patients with sepsis within the first 3 days (7). However, our findings also showed that LVEF might be failing to detect subtle changes in patients with septic myocardial dysfunction within 3 days. The assessment of LVEF by echocardiography in hemodynamically unstable patients with sepsis is indispensable, but it highly depends on the patients' hemodynamic and volume status. The results of this study are in line with the observations by Ronaldo et al., which showed that a low LVEF is neither a sensitive nor a specific predictor of mortality (17).

Our findings demonstrate that LVGLS could detect changes in myocardial performance early in patients with septic myocardial dysfunction within 3 days. STE is considerably less susceptible to changes in preload or afterload because speckle tracking allows for the detection of complex tissue deformation in the myocardium in opposition to measuring the simple displacement. Therefore, the strain measurements resulting from STE are considered to be better correlated with myocardial function than traditional echocardiographic measurements. LVGLS has been thought to be sensitive for detecting the early changes in LV function because the subendocardial fibers, which are the most sensitive to disease, are aligned longitudinally (18).

Our results revealed that 2D LVGLS reduced on the first day, was lowest on the third day, and then gradually improved by the seventh to the tenth day. In clinical practice, LV function in septic cardiomyopathy has not been clearly expounded (19). Previous studies demonstrated a reduced LV function in 30% to 60% of patientswith septic shock (20, 21) and reversibility (22, 23). In a study of 55 patients with septic shock, De Geer et al. found that GLS was reduced within 3 days in patients with sepsis (24). Our study expanded the prior observations by indicating the serial changes in LV myocardial function within 10 days in septic cardiomyopathy. In our study, 3D and 2D STE strains were lowest on the third day and gradually improved on the seventh to the tenth day but did not fully recover to normality. The results of this research support the idea that myocardial dysfunction during septic shock could be described as a state of left ventricular depression, revealing reversibility on remissions (25).

### 3D-STE in patients with sepsis and septic shock

3D echocardiographic measurements are currently recommended for patients with good image quality based on the Consensus of the European Association of Cardiovascular Imaging (EACVI)/ American Society of Echocardiography (ASE) (11). In the intensive care clinical setting, an accurate assessment of LV function is very important. Given that cardiac motion involves 3D movement, 2D STE might lead to the “disappearance” of some strain values from the 2D view by through-plane motion. Consequently, 3D STE can be used to accurately evaluate the natural 3D myocardial movement of the entire left ventricle. The advantage of 3D STE over 2D STE has been demonstrated extensively in healthy subjects and patients with various cardiovascular diseases (26–29), but the application value of 3D STE in patients with sepsis is less well investigated.

We found for the first time that 3D GLS initially decreased and then gradually increased in patients with sepsis and septic shock. The reduced myocardial contractility causes a decrease in 3D GLS, which reflects the progression of sepsis. Our results also highlight the feasibility of 3D STE for serial evaluation in this clinical setting, consistent with the study of Orde et al. (30). The superiority of 3D GLS over 2D GLS may be because it could rely on the minimization of errors independent of LV geometric assumptions. Considering that 3D STE provides more comprehensive details on myocardial performance, it has been proposed as a trial of choice in guidelines for monitoring asymptomatic cardiotoxicity (31–33). Therefore, the present study may have clinical significance in that 3D and 2D STE could provide highly useful and clinically relevant information in sepsis. We reckon that 3D STE for evaluating improvement in LV function in sepsis and septic shock is encouraging.

Our finding showed excellent reproducibility for 3D STE. This result has important clinical implications because it allows us to identify actual changes in LV function. Our observation reinforces and expands previous research by demonstrating the similar value of 3D STE and 2D STE parameters for the serial assessment of LV function in patients with sepsis and septic shock. These results can be particularly attractive in sepsis and septic shock. As a new technology, 3D STE appears promising. However, further clinical validation is needed to determine whether 3D STE is superior to 2D STE.

### Clinical implications

Our results suggest that, for patients with sepsis-induced cardiac dysfunction, future studies could rely more on GLS to judge the severity of the disease and assess response to therapy. 3D and 2D strain imaging, a helpful tool to monitor the evolution of myocardial deformation, will provide clinicians with a useful additional imaging parameter to facilitate the assessment of patients with subtle septic myocardial dysfunction (34).

### Limitations

First, it was a single-center study limited to the ICU. Therefore, the sample size of this present study was relatively small. Second, our study focused on LV myocardial function in patients with sepsis and septic shock within a 10-day period. Thus, future studies need to investigate the changes in LV function in the long term. Third, STE is dependent on image quality. We excluded participants with poor image quality and arrhythmias. Moreover, the study results only apply to tests that use the same post-processing analysis platform. We are also unaware of the feasibility of 2D and 3D STE analyses in this difficult setting. Therefore, the generalizability of our findings is limited. Fourth, 3D STE itself is hindered by low temporal resolution. Temporal resolution, sector size, and width are likely to evolve and improve in the future (35). Finally, the lack of clear and unified diagnostic criteria for LV myocardial impairment in patients with sepsis and septic shock is the fundamental reason restricting research in this field.

## Conclusion

Patients with sepsis or septic shock demonstrated gradual improvements in LV 3D and 2D STE parameters during ICU admission, but their myocardial function did not fully recover to normality on the 10^th^ day. 3D and 2D STE could provide highly useful and clinically relevant information for quantifying LV function and serial follow-up of patients with sepsis or septic shock.

## Data availability statement

The raw data supporting the conclusions of this article will be made available by the authors, without undue reservation.

## Ethics statement

The study protocols were approved by the Institutional Ethical Committee of the Union Hospital, Tongji Medical College, Huazhong University of Science and Technology, Wuhan, China (2021-S047). The patients/participants provided their written informed consent to participate in this study. Written informed consent was obtained from the individual(s) for the publication of any potentially identifiable images or data included in this article.

## Author contributions

XY, YL, JL, and TZ contributed equally to this manuscript. YZ, WS, CS, and JM prepared Tables 1–5 and Figures 1–3. LZ, YS, and MX critically reviewed the manuscript for important intellectual content. All authors approved it for publication. All authors listed made a substantial, direct, and intellectual contribution to the work.

## Funding

This work was supported by the National Natural Science Foundation of China (Grant Nos. 81727805 and 81922033), the Key Research and Development Program of Hubei (Grant Nos. 2020DCD015 and 2021BCA138), and Shenzhen Science and Technology under Grants (SGDX20190917094601717 and JCYJ20210324141216040).

## Conflict of interest

The authors declare that the research was conducted in the absence of any commercial or financial relationships that could be construed as a potential conflict of interest.

## Publisher's note

All claims expressed in this article are solely those of the authors and do not necessarily represent those of their affiliated organizations, or those of the publisher, the editors and the reviewers. Any product that may be evaluated in this article, or claim that may be made by its manufacturer, is not guaranteed or endorsed by the publisher.

## References

[1] Angus DC van der Poll T. Severe sepsis and septic shock. N Engl J Med. (2013) 369:840–51. 10.1056/NEJMra1208623 23984731

[2] Rudd KE Johnson SC Agesa KM Shackelford KA Tsoi D Kievlan DR. Global, regional, and national sepsis incidence and mortality, 1990–2017: analysis for the global burden of disease study. Lancet. (2020) 395:200–11. 10.1016/S0140-6736(19)32989-7 31954465 PMC6970225

[3] Cuthbertson BH Elders A Hall S Taylor J MacLennan G Mackirdy F. Mortality and quality of life in the five years after severe sepsis. Crit Care. (2013) 17:R70. 10.1186/cc12616 23587132 PMC4057306

[4] Beesley SJ Weber G Sarge T Nikravan S Grissom CK Lanspa MJ. Septic Cardiomyopathy. Crit Care Med. (2018) 46:625–34. 10.1097/CCM.0000000000002851 29227368

[5] Vallabhajosyula S Gillespie SM Barbara DW Anavekar NS Pulido JN. Impact of new-onset left ventricular dysfunction on outcomes in mechanically ventilated patients with severe sepsis and septic shock. J Intensive Care Med. (2018) 33:680–86. 10.1177/0885066616684774 28553776

[6] Vallabhajosyula S Shankar A Vojjini R Cheungpasitporn W Sundaragiri PR DuBrock HM. Impact of right ventricular dysfunction on short-term and long-term mortality in sepsis: a meta-analysis of 1,373 patients. Chest. (2021) 159:2254–63. 10.1016/j.chest.2020.12.016 33359215 PMC8579312

[7] Aneman A Vieillard-Baron A. Cardiac dysfunction in sepsis. Intensive Care Med. (2016) 42:2073–6. 10.1007/s00134-016-4503-4 27544139

[8] Seymour CW Liu VX Iwashyna TJ Brunkhorst FM Rea TD Scherag A. Assessment of clinical criteria for sepsis: for the third international consensus definitions for sepsis and septic shock (sepsis-3). JAMA. (2016) 315:762–74. 10.1001/jama.2016.0288 26903335 PMC5433435

[9] Hollenberg SM Singer M. Pathophysiology of sepsis-induced cardiomyopathy. Nat Rev Cardiol. (2021) 18:424–34. 10.1038/s41569-020-00492-2 33473203

[10] Eguia E Bunn C Kulshrestha S Markossian T Durazo-Arvizu R Baker MS. Trends, cost, and mortality from sepsis after trauma in the united states: an evaluation of the national inpatient sample of hospitalizations, 2012–2016. Crit Care Med. (2020) 48:1296–303. 10.1097/CCM.0000000000004451 32590387 PMC7872079

[11] Lang RM Badano LP Mor-Avi V Afilalo J Armstrong A Ernande L. Recommendations for cardiac chamber quantification by echocardiography in adults: an update from the American society of echocardiography and the European association of cardiovascular imaging. J Am Soc Echocardiogr. (2015) 28:1–39e14. 10.1016/j.echo.2014.10.003 25559473

[12] Murad MH Geske JB. Jentzer JC. Global longitudinal strain using speckle-tracking echocardiography as a mortaly predictor in sepsis: a systematic review. J Intensive Care Med. (2019) 34:87–93. 10.1177/0885066618761750 29552957

[13] Evans L Rhodes A Alhazzani W Antonelli M Coopersmith CM French C. Surviving sepsis campaign: international guidelines for management of sepsis and septic shock 2021. Intensive Care Med. (2021) 47:1181–247. 10.1097/CCM.0000000000005337 34599691 PMC8486643

[14] Otto CM Nishimura RA Bonow RO Carabello BA Erwin JP Gentile F. 2020 ACC/AHA guideline for the management of patients with valvular heart disease. J Am Coll Cardiol. (2021) 77:e25–197. 10.1016/j.jacc.2020.11.018 33342587

[15] Sugimoto T Robinet S Dulgheru R Bernard A Ilardi F Contu L. Echocardiographic reference ranges for normal left atrial function parameters: results from the EACVI NORRE study. Eur Heart J Cardiovasc Imaging. (2018) 19:630–8. 10.1093/ehjci/jey018 29529180

[16] Manish Bansal Ravi R. Kasliwal. How do I do it? Speckle-tracking echocardiography. Indian Heart J. (2013) 65:117–23. 10.1016/j.ihj.2012.12.004 23438628 PMC3860973

[17] Sevilla Berrios RA O'Horo JC Velagapudi V Pulido JN. Correlation of left ventricular systolic dysfunction determined by low ejection fraction and 30-day mortality in patients with severe sepsis and septic shock: a systematic review and meta-analysis. J Crit Care. (2014) 29:495–9. 10.1016/j.jcrc.2014.03.007 24746109

[18] Vignon P Huang SJ. Global longitudinal strain in septic cardiomyopathy: the hidden part of the iceberg? Intensive Care Med. (2015) 41:1851–3. 10.1007/s00134-015-3962-3 26183488

[19] Lanspa MJ Olsen TD Wilson EL Leguyader ML Hirshberg EL Anderson JL. A simplified definition of diastolic function in sepsis, compared against standard definitions. J Intensive Care. (2019) 7:14. 10.1186/s40560-019-0367-3 30820322 PMC6381727

[20] Weng L Liu YT Du B Zhou JF Guo XX Peng JM. The prognostic value of left ventricular systolic function measured by tissue Doppler imaging in septic shock. Crit Care. (2012) 16:R71. 10.1186/cc11328 22554063 PMC3580613

[21] Bergenzaun L Gudmundsson P Ohlin H During J Ersson A Ihrman L. Assessing left ventricular systolic function in shock: evaluation of echocardiographic parameters in intensive care. Crit Care. (2011) 15:R200. 10.1186/cc10368 21846331 PMC3387642

[22] Vieillard Baron A Schmitt JM Beauchet A Augarde R Prin S Page B. Early preload adaptation in septic shock? A transesophageal echocardiographic study. Anesthesiology. (2001) 94:400–6. 10.1097/00000542-200103000-00007 11374597

[23] Bouhemad B Nicolas-Robin A Arbelot C Arthaud M Feger F Rouby JJ. Acute left ventricular dilatation and shock-induced myocardial dysfunction. Crit Care Med. (2009) 37:441–7. 10.1097/CCM.0b013e318194ac44 19114917

[24] De Geer L Engvall J Oscarsson A. Strain echocardiography in septic shock - a comparison with systolic and diastolic function parameters, cardiac biomarkers and outcome. Crit Care. (2015) 19:122. 10.1186/s13054-015-0857-1 25882600 PMC4374340

[25] Parrillo JE Burch C Shelhamer JH Parker MM Natanson C Schuette W. A circulating myocardial depressant substance in humans with septic shock. Septic shock patients with a reduced ejection fraction have a circulating factor that depresses in vitro myocardial cell performance. J Clin Invest. (1985) 76:1539–53. 10.1172/JCI112135 4056039 PMC424125

[26] Nagata Y Takeuchi M Wu VC Izumo M Suzuki K Sato K. Prognostic value of LV deformation parameters using 2D and 3D speckle-tracking echocardiography in asymptomatic patients with severe aortic stenosis and preserved LV ejection fraction. JACC Cardiovasc Imaging. (2015) 8:235–45. 10.1016/j.jcmg.2014.12.009 25682511

[27] Trache T Stöbe S Tarr A Pfeiffer D Hagendorff A. The agreement between 3D, standard 2D and triplane 2D speckle tracking: effects of image quality and 3D volume rate. Echo Res Pract. (2014) 1:71–83. 10.1530/ERP-14-0025 26693303 PMC4676467

[28] Saito K Okura H Watanabe N Hayashida A Obase K Imai K. Comprehensive evaluation of left ventricular strain using speckle tracking echocardiography in normal adults: comparison of three-dimensional and two-dimensional approaches. J Am Soc Echocardiogr. (2009) 22:1025–30. 10.1016/j.echo.2009.05.021 19556106

[29] Lv Q Sun W Wang J Wu C Li H Shen X. Evaluation of biventricular functions in transplanted hearts using 3-dimensional speckle-tracking echocardiography. J Am Heart Assoc. (2020) 9:e015742. 10.1161/JAHA.119.015742 32370590 PMC7660853

[30] Orde S Slama M Stanley N Huang S McLean A. Feasibility of biventricular 3D transthoracic echocardiography in the critically ill and comparison with conventional parameters. Crit Care. (2018) 22:198. 10.1186/s13054-018-2133-7 30121088 PMC6098822

[31] Kalam K Otahal P Marwick TH. Prognostic implications of global LV dysfunction: a systematic review and meta-analysis of global longitudinal strain and ejection fraction. Heart. (2014) 100:1673–80. 10.1136/heartjnl-2014-305538 24860005

[32] Hai PA-OX Phuong LL Dung NM Hoa LTV Quyen DV Chinh NX. Subclinical left ventricular systolic dysfunction in patients with septic shock based on sepsis-3 definition: a speckle-tracking echocardiography study. Crit Care Res Pract. (2020) 2020:6098654. 10.1155/2020/6098654 33014463 PMC7525316

[33] Sanfilippo F Huang S Herpain A Balik M Chew MS Clau-Terré F. The PRICES statement: an ESICM expert consensus on methodology for conducting and reporting critical care echocardiography research studies. Intensive Care Med. (2021) 47:1–13. 10.1007/s00134-020-06262-5 33275163

[34] Ehrman RR Moore SC Favot MJ Akers KG Gallien JZ Welch RD. Scientific letter to the editor: need for a definitive study of global longitudinal strain for prognostication in septic cardiomyopathy. J Am Soc Echocardiogr. (2019) 32:549–52 e3. 10.1016/j.echo.2018.12.005 30738639

[35] Balasubramanian S Punn R Smith SN Houle H Tacy TA. Left ventricular systolic myocardial deformation: a comparison of two- and three- dimensional echocardiography in children. J Am Soc Echocardiogr. (2017) 30:974–83. 10.1016/j.echo.2017.06.006 28802483

